# Episodic tremor and slip silently invades strongly locked megathrust in the Nankai Trough

**DOI:** 10.1038/s41598-019-45781-0

**Published:** 2019-06-25

**Authors:** Masayuki Kano, Aitaro Kato, Kazushige Obara

**Affiliations:** 1 Graduate School of Science, Tohoku University, 6-3, Aramaki-aza-aoba, Aoba-ku, Sendai 980-8578 Japan; 2 Earthquake Research Institute, The University of Tokyo, 1-1-1, Yayoi, Bunkyo-ku, Tokyo 113-0032 Japan

**Keywords:** Seismology, Tectonics

## Abstract

Recent seismic and geodetic observations in subduction zones have revealed that slow earthquakes have preceded some large earthquakes. Characterization of slow earthquakes and their relation to large earthquakes provides important clues to constrain a wide spectrum of slip rates on tectonic faults. Here, we report new evidence of a slow slip transient at the downdip edge of the strongly locked seismogenic zone in the western Nankai Trough in southwest Japan. This slow slip transient was excited during an episodic tremor and slip at the downdip extension of the locked zone. Through this triggering, the frequent occurrence of the deep episodic tremor and slip invades the strongly locked megathrust zone and may intermittently increase the probability of large earthquakes in the Nankai Trough.

## Introduction

Slow slip transients on faults may be connected to the occurrence of megathrust earthquakes along plate subduction zones^1^. Recent geodetic observations have clarified a decadal-scale transient slip in the deeper part of the strongly locked seismogenic zone before the 2011 Tohoku-oki earthquake^2–4^. In addition, monthly-scale slow slip events (SSEs) in the vicinity of the dynamic rupture initiation point have preceded some large subduction earthquakes in Tohoku, Mexico, and Chile^5–9^. Such transient slips represent slow accommodation processes of cumulative stress around the fault that simultaneously transfers stress to the nearby seismogenic zone. Numerical simulations have inferred that repetition of stress transfer driven by sequences of transient slips gradually invades the locked zone in the latter half of interseismic intervals^10,11^. Therefore, refined characterization of the spatio-temporal evolution of slow slip transients within the locked zone is extremely important for our fundamental understanding of megathrust earthquakes.

**Figure 1:**
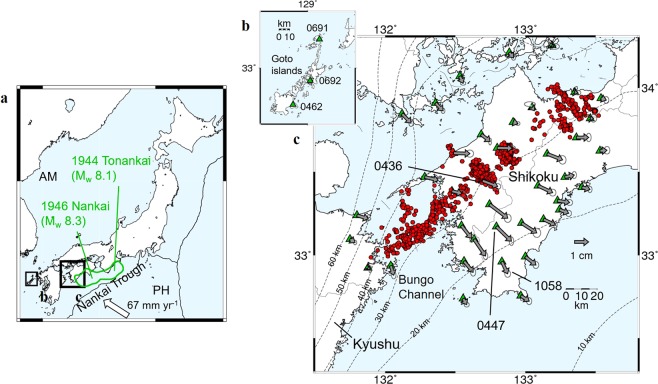
Tectonic settings and stacked GNSS displacements. (**a**) Tectonic setting of western Shikoku, in southwestern Japan. The Philippine Sea plate (PH) is subducting beneath the Amurian plate (AM) at a velocity of ~67 mm yr^−1^, indicated by a white arrow^12^. The light green line indicates the source areas of the 1944 Tonankai and 1946 Nankai earthquakes^30^. Rectangles indicate the map insets shown in (**b**,**c**). (**b**) Reference GNSS stations in the Goto islands (green triangles). (**c**) Stacked surface displacements at GNSS stations in western Shikoku (green triangles) with 2-σ observation errors shown by the ellipsoids. Black arrows show the observed displacement vectors. Red dots indicate LFE locations. The dashed lines indicate depth contours of the upper surface of the subducting PH with an interval of 10 km^41–43^. Stacked and individual GNSS time series at three stations indicated by 4 digits and all the other stations are shown in Figs 2 and S1, respectively.

Here, we focus on the Nankai subduction zone beneath western Shikoku Island in southwest Japan (Fig. 1a,c), where the Philippine Sea plate (PH) is subducting beneath the Amurian plate (AM) at a velocity of ~67 mm yr^−1^ ^12^. In this region, moment magnitude (M_w_) ~8 class earthquakes are known to occur at intervals of 90–200 years^13^; more than 70 years have already passed since the previous 1944 Tonankai and 1946 Nankai earthquake sequences. Both onshore and offshore geodetic observations have clarified that the plate boundary fault has been strongly locked at depths shallower than ~20 km^14,15^. SSEs have been well documented at the downdip extension of the strongly locked seismogenic zone using tilt/strain meters and Global Navigation Satellite System (GNSS) observations^16–20^. These SSEs are classified into two types according to their slip durations^1^. First, a well-documented M_w_ ~7 class long-term SSEs (L-SSEs) occurred with a recurrence interval of ~6 years and durations of approximately one year in the Bungo Channel^19,20^. On the eastern side of the Bungo Channel, L-SSEs that release an equivalent seismic moment of M_w_ 6.0–6.3 occurred in 2004–2006 and 2011–2013 at depths of 20–30 km just below the strongly locked seismogenic zone^18^. Such L-SSEs often induce an increase in deep non-volcanic tremors on the updip side of the tremor band (~30–35 km)^18,21^. Second, short-term SSEs (S-SSEs), that lasts for weeks and frequently occur at depths of ~30–35 km with an interval of several months^16,17^, are concordant with deep non-volcanic tremors; these are referred to as episodic tremor and slip (ETS) events^22^.

**Figure 2:**
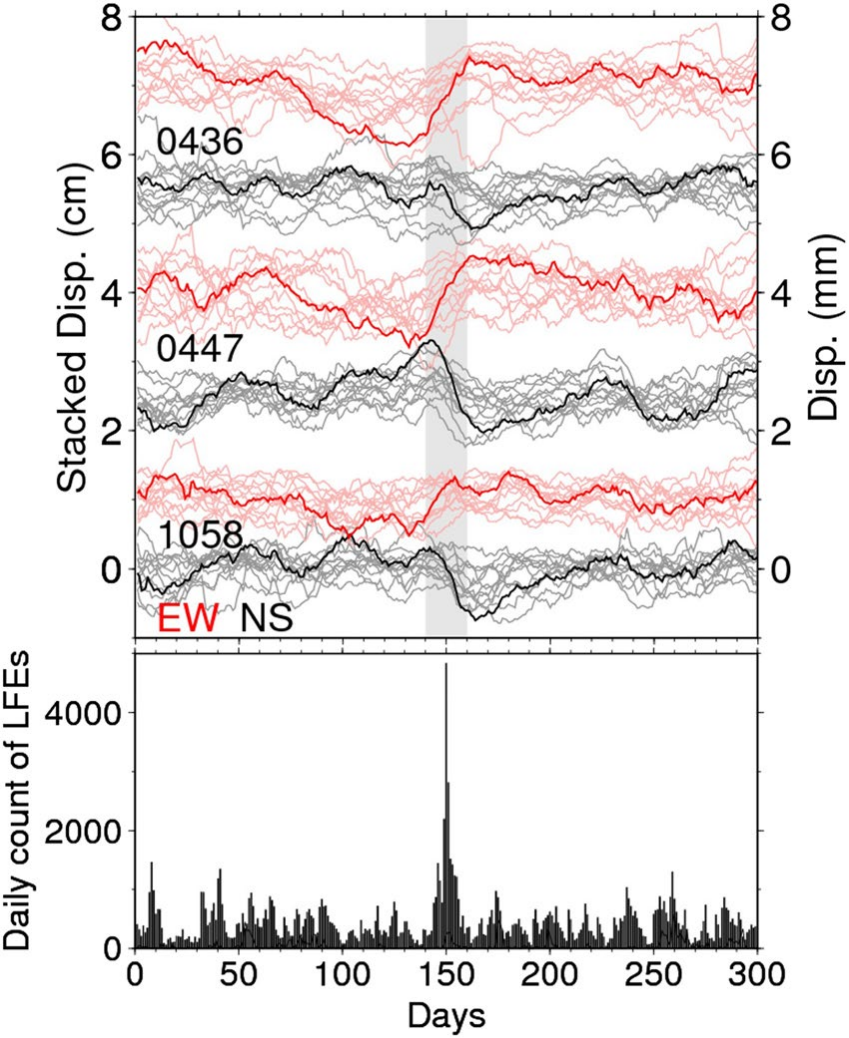
Stacked GNSS time series. (Top) Red and black lines indicate the east-west (EW) and north-south (NS) components of the stacked GNSS time series cumulated for the 12 S-SSEs at stations 0436, 0447, and 1058 (Fig. 1c), respectively. Light red and gray lines are the EW and NS components of the GNSS time series during each ETS event. (Bottom) Time series of daily count of LFEs cumulated for the 12 S-SSEs.

However, the connection between downdip SSEs and updip locked seismogenic zones is still elusive. Here, we report on a slow slip transient at the downdip edge of the strongly locked seismogenic zone during downdip ETS events, newly detected by inverting the stacked GNSS time series data. These stacked time series are calculated by summing up the detrended time series from each S-SSE that occurred from 2004 to 2009, with the assumption that the slip rate of S-SSEs correlates well with the number of deep low frequency earthquakes (LFEs)^23^ (Table S1, Figs 2 and S1; see Methods). This new finding provides a possible direct link between ETS events and unlocking at the bottom of the locked seismogenic zone.

## Stacked GNSS Signals

Based on continuous data from GNSS stations and the number of LFEs observed, we derived the cumulative stacked time series for 12 sequences of S-SSEs detected by tiltmeters^16^ and/or GNSS^17^ between April 2004 and March 2009 (Table S1; see Methods). Figure 2 shows the stacked time series at stations 0436, 0447, and 1058, located in western Shikoku, demonstrating ~0.4–1.1 cm eastward and ~0.4–1.0 cm southward motions of these stations between days 140 and 160 (10 days before and after the reference date, day 150). Stacked time series obtained at the other remaining 36 stations are summarized in Fig. S1. During this period, almost all stations exhibited coherent southeastward movement (Figs 1c and S1), with maximum southeastward displacements of ~1.4–1.6 cm near station 0447. These surface displacements occurred in roughly the opposite direction of PH plate motion relative to the AM, indicating a slow slip on the plate interface.

## Cumulative Slip Distribution for 12 S-SSEs

**Figure 3:**
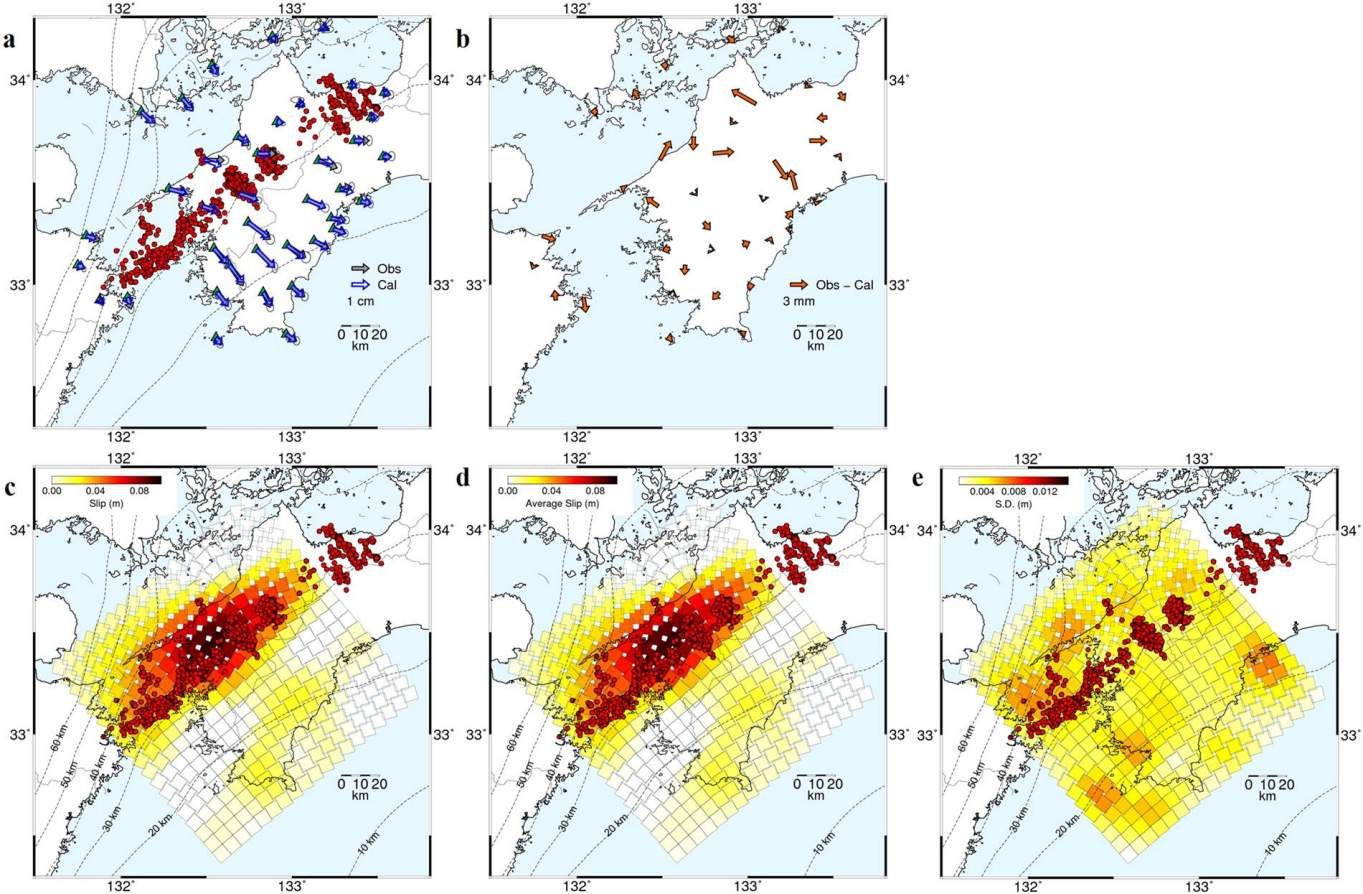
Calculated vectors and slip models. (**a**) Comparisons between the observed (black) and the calculated (blue) displacement vectors and (**b**) the residuals between the two vectors. The ellipsoids indicate the 2-σ observation errors. (**c**) Cumulative slip distribution when all 39 GNSS stations are used. (**d**) Slip distribution averaged for 100 cases obtained by using 35 randomly selected stations and (**e**) its standard deviation. Red dots indicate the LFE locations. The dashed lines are depth contours of the upper surface of the subducting PH at an interval of 10 km^41–43^.

The cumulative slip distribution (Fig. 3c) shows that there are two major slip patches exceeding an estimation error of 1-σ (Fig. S2; see Methods). A patch with large slip displacement is elongated along strike at depth of ~35 km with a maximum slip of ~9.2 cm, located in a slightly deeper part of a belt-like zone of LFE/tremor sources. This area is consistent with the source area hosting multiple S-SSEs from 2004–2009 based on previous geodetic studies^16,17^. In addition, a relatively small slip displacement (~1.5–2.2 cm) slightly greater than slip estimation errors (~1.1–1.4 cm) (Fig. S2), can be identified on the shallower part of the plate interface at a depth of ~20 km. The existence of this shallow slow slip is revealed by inverting the stacked GNSS surface displacements through the improvement of the signal-to-noise ratio.

To validate the estimated slip, we conducted slip inversion for 100 cases using the displacement vectors at 35 randomly selected GNSS stations, and obtained the average slip distribution and its standard deviation (Fig. 3d,e). The average slip distribution was quite similar to that estimated using all GNSS stations (Fig. 3c). The standard deviations for 100 cases in deep and shallow slip patches were estimated to be 0.2–0.7 cm and 0.2–0.4 cm, respectively (Fig. 3e), indicating that the estimated slip was significant in both patches. We confirmed that slip in both patches was meaningful by conducting a similar test of 31 randomly selected GNSS stations, although the standard deviations were larger (Fig. S3). Surface displacements calculated from a shallower slip exceeding the 1-σ estimation error (Fig. S2b) were not so large and at most comparable to the 2-σ observation errors (see Methods) at several stations located in the southern part of the region (Fig. S4). However, the misfit between the observed and calculated displacement vectors at these stations became worse when we did not allow fault slip along the shallow subfaults (Figs S5b and 3b; see Supplementary Note). In addition, we have conducted additional slip inversions to investigate the effect(s) of the plate geometry model, elastic properties, and slip direction on shallow slip. (Table S2 and Figs S6–S8; see Supplementary Note). Based on these different sorts of tests, we concluded that slow slip transients occurred not only in deep areas (~35 km), but also in a relatively shallow region (~20 km) of western Shikoku, close to the southern coast. However, the amount of shallow slip is as small as the 1- to 2-σ estimation error level, indicating that the detection is weak, and the details are not well resolved. We thus need to stack more data to enhance the signal to noise ratio of the shallow slip in future.

Our analysis revealed two slow slip patches located in deep and shallow areas of the plate interface (Fig. 3c). Seismic moments for deep and shallow patches calculated from the estimated slip exceeded the estimation error, assuming a rigidity of 40 GPa, were 1.31 × 10^19^ Nm and 8.62 × 10^17^ Nm, respectively, equivalent to M_w_ of 6.68 and 5.89, respectively. The seismic moment for each S-SSE in the deep patch was also estimated in previous studies^16,17^. The total seismic moment expected from the previous S-SSE sequences was 1.66 × 10^19^ Nm (M_w_ 6.75). In this calculation, we used the average seismic moment for the S-SSEs detected by both tiltmeter^16^ and GNSS^17^ data because there were no systematic differences in magnitudes calculated from these results^17^. The moment magnitude estimated in our analysis is comparable to that expected from previous studies^16,17^, and thus the adopted method is useful for understanding the overall cumulative slip characteristics for S-SSEs.

## Slow Slip Transient at the Bottom of the Strongly Locked Megathrust

**Figure 4:**
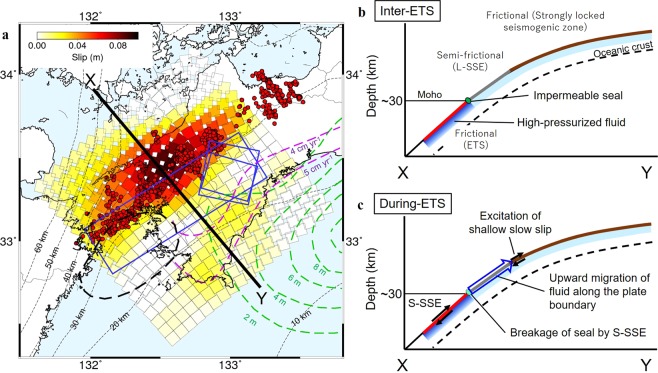
Schematic illustration of the plate boundary in western Shikoku. (**a**) Spatial relation among cumulative slip distribution of S-SSEs (as in Fig. 3c), L-SSEs (blue rectangles^18^; black dotted line^20^), slip deficit rate (purple lines^14^) and coseismic slip distribution in the 1946 Nankai earthquake (green lines^30^). Red dots indicate LFE locations. The black dashed lines are the depth contours of the upper surface of the subducting PH with an interval of 10 km^41–43^. The vertical profiles in the black line (X-Y) are illustrated in (**b**,**c**). (**b**,**c**) Schematic illustration of the vertical cross-section of X-Y in the inter-SSE and ETS periods, respectively. Red, blue, and brown lines correspond to the ETS, L-SSE and locked seismogenic zones, respectively. The green rectangle indicates a seal that confines fluid below in (**b**), the inter-SSE period and is broken in (**c**), the ETS period.

The spatial relation between ETS and locked seismogenic zones was explained by a thermo-petrological model that describes the frictional and viscous regimes of fault slip behavior^24^. In this model, the ETS zone acts in response to frictional strength and occurs in an isolated zone where pore fluid pressure is locally elevated around the mantle wedge corner. The deep slip patch imaged by the present model (Fig. 3c) corresponds to this well-described ETS zone in a frictional regime. In the case of Nankai^18^ and Mexico^23,25^ subduction zone, between the deep ETS and locked seismogenic zones there exists a transitional zone that exhibits a mixed (‘semi-frictional’) behavior of both friction and viscous deformation. This transitional zone corresponds to a depth of ~25 km, where both relatively small L-SSEs (equivalent to M_w_ 6.0–6.3) and large Bungo L-SSEs (equivalent to M_w_ ~7) have been detected in western Shikoku via GNSS (Fig. 4a)^18–20^. L-SSEs are the manifestation of semi-frictional behavior, and the existence of the semi-frictional region may prevent the quick transfer of stress change caused by ETS events in the seismogenic zone^24^. In contrast with Gao and Wang’s model^24^, the most striking feature revealed by this study is that the small slow slip transient occurs even at the downdip edge of the strongly locked seismogenic zone.

## Plausible Mechanism for the Simultaneous Occurrence of Deep and Shallow Slow Slip Transients

Another important feature is that transient slip in the shallow patch occurs simultaneously with the deep ETS beyond the spatial gap hosting small L-SSEs in the semi-frictional regime. Note that the GNSS data was averaged using a moving window of 20 days and therefore slip occurrence times in both patches may differ for, at most, a few tens of days. A possible cause for this almost simultaneous occurrence of slow slip transients with the spatial gap is an upward fluid migration along the plate boundary, from the deep ETS zone to the downdip edge of the locked seismogenic zone^26,27^. Tanaka *et al*.^26^ proposed a fluid migration model for the Tokai L-SSE zone (in central Japan) to qualitatively explain the observed gravity change during the SSE. In their model, highly-pressurized fluid released from the subducting oceanic crust is confined along the plate boundary by both impermeable seals and an impermeable cap rock at the bottom of the overriding plate before the occurrence of SSE (Fig. 4b). When the SSE initiates, the impermeable seals may be broken due to shear failure, and the fluids begin to flow along the plate interface to shallower depths (Fig. 4c). If fluid migrates towards shallower depths, it may reduce effective normal stress through the local increase in fluid pressure, enhancing shallow slow slip. Although the time lag required for fluid migration in this model was estimated to be on the order of years, more rapid fluid migration on a daily to monthly time scale has been inferred from numerical simulations of pore-pressure waves along subduction zone^28^ and geochemical analysis of Lithium-diffusion^29^. Therefore, this model can explain the almost simultaneous occurrence of the shallow small and deep large slow slip transients detected in western Shikoku. However, the migrated fluid may not change the shear strength in the semi-frictional regime sandwiched between the two slow slip patches because viscous deformation is less sensitive to variations in effective normal stress^24^.

Similar behavior, i.e., small SSEs would occur during large SSEs, was inferred during the 2006 SSE beneath Guerrero, Mexico, by Frank *et al*.^25^ which decomposed the surface displacement time series utilizing LFE activities as an indicator of slow slip transients. This model for the mechanism of the L-SSE is not directly applied to our case; however, more elaborate studies regarding the interplay among small and large SSEs should be needed.

## Role of Shallow Slow Slip Transients on Megathrust Rupture

The shallow slow slip transient is adjacent to the strongly locked zone with a slip deficit rate greater than 5 cm yr^−1^ within the source region of the 1946 Nankai earthquake^30^ (Fig. 4a). The slip deficit rate in the shallow patch is estimated to be roughly 4 cm yr^−1^ from onshore and offshore geodetic observations^14,15^ (Fig. 4a). The present study estimates that roughly 1.5–2.2 cm of shallow slip occurred over five years. In addition, the shallow patch exhibited a few centimeters of slip from 2013–2016 when SSEs appeared to migrate from the east, off southern Kyushu, to the Bungo Channel^31^. These cumulative slow slip transients for approximately 8 years correspond to ~5–6 mm yr^−1^ at the average slip rate. Considering the plate convergence rate of the PH relative to the AM (~67 mm yr^−1^)^12^, ~8–9% of the strain energy accumulated due to plate convergence is accommodated by slow slip transients in the downdip edge of the locked seismogenic zone. Such slow slip transients may explain the relatively lower slip deficit rate of 4–5 cm yr^−1^ in this region compared to the updip strongly locked seismogenic zone (>5 cm yr^−1^) (Fig. 4a)^14^.

The transient slip in the shallow patch causes direct stress loading onto the locked seismogenic zone that will mainly host megathrust dynamic ruptures. It has been reported that precursory slow slip transients at the downdip or updip portion of the firmly locked area may have promoted large earthquakes in subduction zones^5–9^. Although there have been no large earthquakes along the Nankai Trough after the shallow slow slip transient detected in this study, such slow slips can temporarily increase the probability of triggering large earthquakes, especially when the accumulated stress in nearby strongly locked segments is close to failure^32^. In addition, the shallow slow slip transient occurs simultaneously with the downdip ETS events. Hence, S-SSEs in the ETS zone transfer stress to the locked seismogenic zone via the excitation of shallow slow slip transients.

Our results indicate that episodic slow slip transients occur at the bottom of the strongly locked seismogenic zone, which has previously been considered to have little interseismic slip. Numerical simulations demonstrate that coupling in the deeper part of the seismogenic zone continues to weaken, especially in the latter half of the interseismic period, due to stress accumulation caused by repeated slow slip transients in the adjacent downdip portion^10^. Although it is not certain that slow slip transients directly result in megathrust earthquakes^33^, they transfer stress to the megathrust earthquake zone and play an important role in stress accumulation. Therefore, careful monitoring of the unlocking process at the bottom of the locked seismogenic zone is essential for quantitatively evaluating stress transfer in the megathrust earthquake zone.

## Methods

### Detection of S-SSE signals in western Shikoku

#### Methodology

We used a method to detect small signals from S-SSEs by stacking GNSS time series data to increase the signal-to-noise ratio^23^. In this method, low frequency earthquakes (LFEs), which are considered to be isolated signals of tremors^34^, are adopted as an indicator of S-SSEs. Assuming that the number of LFEs correlates well with slip rate along the plate interface^35^, the timing of each S-SSE is defined as the time at which the LFE occurrence rate is at its maximum. Based on the timing for each S-SSE, detrended GNSS time series data are stacked for all S-SSEs. Applying this method to the GNSS data in the Guerrero region, Frank *et al*.^23^ successfully extracted signals from multiple S-SSEs and inferred the cumulative spatial distribution of S-SSEs solely from GNSS data. Following this study, we estimated the cumulative spatial distribution of transient slips by stacking the detrended GNSS data in western Shikoku using a dense catalog of LFEs.

#### LFE estimation

To more precisely investigate the spatio-temporal evolution of LFEs, we systematically searched for events with seismograms similar to those observed for a number of well-recorded template LFEs by applying a matched filter technique (MFT)^34,36^. We used continuous three-component velocity seismograms retrieved by a nationwide high-sensitivity seismograph network (Hi-net)^37,38^ between April 2004 and August 2015. As template events, we selected 1,939 LFEs in Shikoku with relatively high signal-to-noise ratios as identified by the Japan Meteorological Agency (JMA). The continuous and template waveforms were preprocessed by using bandpass filtering between 2 and 6 Hz, and decimating the sampling from 100 to 20 Hz. We then extracted a 6.0 s data window, starting 3.0 s prior to the synthetic S-wave arrivals. Synthetic arrivals were calculated using the one-dimensional velocity structure of the JMA. The event detection threshold was set at 9 times the median absolute deviation of the average correlation coefficient calculated over the day of interest.

#### GNSS data analysis

Continuous GNSS data were obtained from GEONET, which has been operated by the Geospatial Information Authority of Japan (GSI). We analyzed daily coordinates in two horizontal components at 39 stations in and around western Shikoku, in southwestern Japan, derived from the GEONET F3 solution^39^ from April 2004 to March 2009. To obtain the relative displacement fixed to the Amurian plate and to remove common mode errors, the daily displacements, averaged over three stations (0462, 0691, and 0692; Fig. 1b) located in the Goto islands, west of Kyushu Island, were removed from the GNSS time series at each station. Then we smoothed each time series by taking 20-day moving averages.

Based on the smoothed time series, we attempted to extract the cumulative displacements due to S-SSEs by stacking the GNSS time series for individual SSEs. During the analysis period, 12 SSEs had already been detected by tiltmeter^16^ and/or GNSS^17^ data. Assuming that the number of LFEs reflects the slip velocity along the plate interface, we used the LFE catalog to define the reference date for each S-SSE when the number of LFEs was at its maximum during the period 5 days before and 15 days after the onset day detected by previous studies. The reference date for each S-SSE was used to stack the smoothed GNSS time series. Then, we detrended the GNSS data using the 300 days, centering the reference date for each S-SSE and respectively stacked over 12 SSEs for each component to obtain the stacked time series (Figs 2 and S1). We extracted the surface displacements from 12 S-SSEs as the difference of the stacked time series between days 140 and 160, which corresponds to 10 days before and after the reference date, respectively (Fig. 1c). The errors of each vector were evaluated from the standard deviations of stacked data from a linear trend, that is, we respectively fitted a linear function to the stacked data from days 50 to 100 and from days 200 to 250, and evaluated the standard deviation of residuals from the best fitting linear line, which was defined as the observation error in each station.

### Inversion procedure

The displacement vectors (Fig. 1c) were inverted to the cumulative slip on the plate interface^40^. We generated a geometry model of the plate interface of the subducting PH from depth contours^41–43^ (Fig. 1c). The model covered the region beneath western Shikoku and was divided into 19 × 19 subfaults in the dip and strike direction such that the area of each subfault was 7.5 × 7.5 km when projected on the Earth’s surface. An elastic response at each GNSS station due to a unit slip at each subfault was calculated using the formulation of Okada^44^, assuming a homogeneous half-space with a Poisson’s ratio of 0.25. The rake angle in each subfault was assumed to be ~60–150° depending on the fault strike direction, so that the slip direction was fixed to be N125E which is opposite direction of the plate convergence of the PH relative to the AM (N55W)^12^. Slip distribution was obtained by minimizing the following function;

1 $$s({\bf{a}})={({\bf{d}}-{\bf{H}}{\bf{a}})}^{{\rm{T}}}{{\bf{E}}}^{-1}({\bf{d}}-{\bf{H}}{\bf{a}})+{\alpha }^{2}{{\bf{a}}}^{{\rm{T}}}{\bf{G}}{\bf{a}},$$

where **d** is the observed displacements, **H** is the matrix consisting of elastic response, **a** is the slip vector to be modeled, **E** is the covariance matrix for the observed displacements, and **G** is the smoothing operator. The first term indicates the misfit between the observed and calculated displacement vectors and the second term is a prior constraint for the slip vector so that the slip for neighboring subfaults in both the strike and dip directions varies smoothly. Furthermore, we assume additional subfaults (which are not shown in the figures) surrounding the model region where the slip was set to be zero so that the amount of slip at the edge of the model region became small. We do not assume non-negative slip. The hyperparameter *α*^2^ that controls the weight of spatial smoothness of slip was objectively determined by minimizing Akaike’s Bayesian information criterion (ABIC)^45^. The estimation errors for the model parameters correspond to diagonal elements of the following error covariance matrix

2 $${\bf{C}}=\frac{s(\hat{{\bf{a}}})}{(N+P-M)}{({{\bf{H}}}^{{\rm{T}}}{{\bf{E}}}^{-1}{\bf{H}}+{\alpha }^{2}{\bf{G}})}^{-1},$$

where $\hat{{\bf{a}}}$ is the optimized model parameter vector, *N* is the number of data, *P* is rank of **G**, and *M* is the number of model parameters.

## Supplementary information

Supporting Information
