# Supplementary material for: Episodic tremor and slip silently invades strongly locked megathrust in the Nankai Trough
Source: Sci Rep. 2019 Jun 25;9:9270. doi: 10.1038/s41598-019-45781-0 (PMC6592932; doi:10.1038/s41598-019-45781-0)
Supplement: Supplementary file 1 — Supporting Information [file 41598_2019_45781_MOESM1_ESM.pdf]

Episodic tremor and slip silently invades strongly locked megathrust in the Nankai Trough

Masayuki Kano[1*], Aitaro Kato[2], Kazushige Obara[2]

[1]Graduate School of Science, Tohoku University, 6-3, Aramaki-aza-aoba, Aoba-ku, Sendai, 980-8578, Japan.

[2]Earthquake Research Institute, The University of Tokyo, 1-1-1, Yayoi, Bunkyo-ku, Tokyo, 113-0032, Japan.

*Correspondence to: masayuki.kano.a3@tohoku.ac.jp

Supplemental note

In this supplemental notes, to demonstrate the robustness of the shallow slip patch, we show dozens of slip models obtained by conducting additional inversions under different sorts of model settings; fault models, plate geometry, elastic properties and slip direction.

We performed an inversion in which the shallower 7 × 19 subfaults were completely removed. The larger fault slip with a maximum amplitude of ~9.5 cm was inferred in the deep patch (Fig. S5c), compared to the slip model shown in the main text (hereafter, refer as the final slip model) (Fig. 3c). The calculated vectors are within or close to the error ellipsoids. However, the value of ABIC was 389.26, which was significantly larger than 236.54 in the final slip model. In fact, the misfit between the observed and calculated displacement vectors became systematically smaller in stations close to the southern coast by introducing the shallow subfaults (Figs. 3b and S5b). Therefore, we conclude that the shallower slip is necessary to better explain the observed vectors in the southern stations.

The final slip model was obtained by assuming one of the plate geometry models[41-43] and a Poisson's ratio of 0.25. Thus, one may argue that the small shallow slip could be biased by the assumed plate geometry and/or elastic properties. To investigate the robustness of the shallow slip dependency on plate geometry and elastic properties, we conducted additional sets of inversion using different plate geometries[46,47] and a plausible range of Poisson's ratio[48] (Table S2). We confirmed that all the inversion results exhibited two slip patches at both shallow and deep depths, which closely resembles the final slip model shown in Fig. 3c, despite any combination of plate geometry and Poisson's ratio. The slip amplitude in the final slip model (Fig. 3c) is the smallest among the three plate geometry models[41-43,46,47], because the depth of the plate interface adopted in the final model is the shallowest. Fig. S6 shows two slip models that exhibit the smallest and largest slip amplitudes in the shallow slip patch where the seismic moments released from each slow slip transient are calculated to be 0.85×10^{18} and 4.69×10^{18}

Nm, respectively (Table S2). To assess the significance of the slip amplitude at the shallow slip patch for these two end-member models, we conducted slip inversions for 100 cases using 35 randomly selected GNSS stations and obtained the average slip distribution and its standard deviation (Fig. S7). Both results indicate that the shallow slip was significant, regardless of different combinations of plate geometries and elastic properties. In addition, seismic wave velocity structure imaged by earthquake tomography has indicated that there is no strong lateral elastic heterogeneity within the overriding plate beneath the western Shikoku region[48].

It may be possible to further consider a three-dimensional crustal structure including surface topography. In the case of the SSEs in New Zealand, the elastic response assuming a homogeneous half-space underestimates the amplitude of shallow fault slip by more than 40% compared to the case that assumes a heterogeneous crustal structure[49,50]. Thus the shallow slow slip transient in the present study is well above the estimation error, if the effect of three-dimensional crustal structure is considered.

Figure S8 summarizes the slip models obtained by changing the slip directions between N105E to N145E with a 10 degree interval (the slip direction in the final slip model was fixed to N125E). These slip models commonly showed a shallow slip patch, although there was a slight difference in its magnitude of slip. This result means that the shallow slip patch was robust with regard to the small change (20 degrees) in slip directions. Combining all of these analyses described above, we conclude that the fault slip in the shallow subfaults is a significant feature.

References:

46. G. Hayes, et al., Slab2, a comprehensive subduction zone geometry model, *Science*, **362**(6410), 58–61 (2018).

47. K. Shiomi, M. Matsubara, Y. Ito, K. Obara, Simple relationship between seismic activity along Philippine Sea slab and geometry of oceanic Moho beneath southwest Japan, *Geophysical Journal International*, **173**(3), 1018–1029 (2008).
48. M. Matsubara, K. Obara, K. Kasahara, High-VP/VS zone accompanying non-volcanic tremors and slow-slip events beneath southwestern Japan, *Tectonophysics*, **472**(1-4), 6-17 (2009).
49. C. A. Williams, L. M. Wallace, Effects of material property variations on slip estimates for subduction interface slow-slip events, *Geophys. Res. Lett.*, **42**(4), 1113-1121 (2015).
50. C. A. Williams, L. M. Wallace, The impact of realistic elastic properties on inversions of shallow subduction interface slow slip events using seafloor geodetic data, *Geophys. Res. Lett.*, **45**, 7462-7470 (2018).

Supplemental Figures and Tables

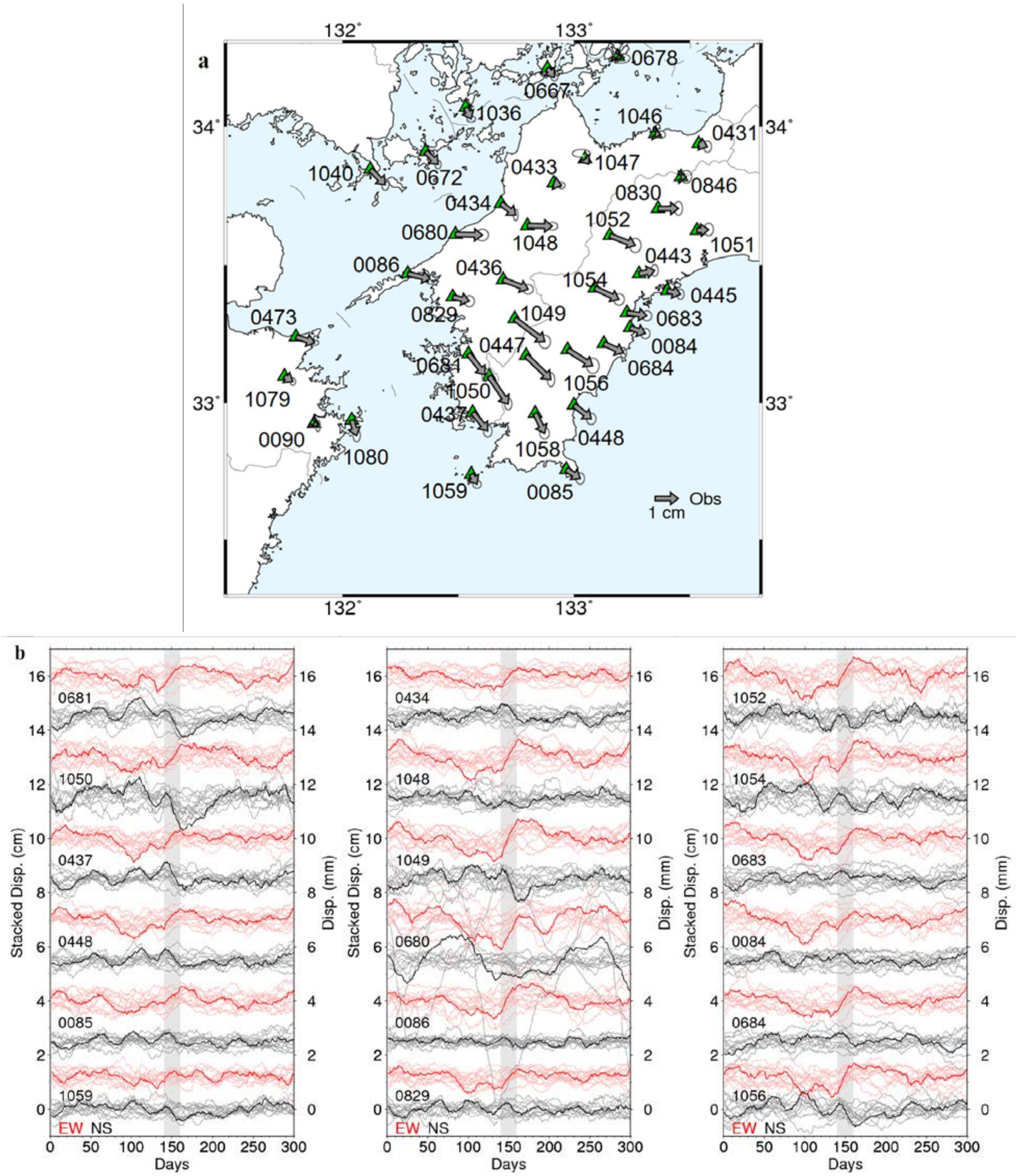

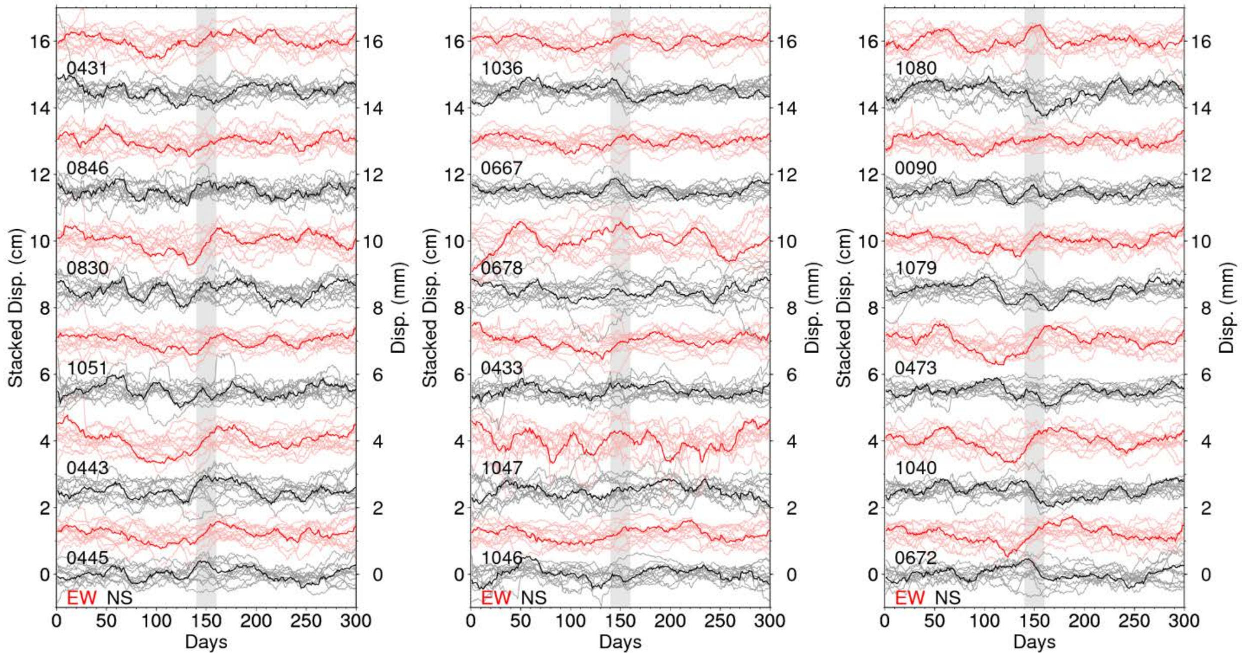

Fig. S1. Stacked GNSS time series for all stations. a, Stacked displacements at GNSS stations in western Shikoku (green triangles) with 2-σ observation errors shown by the ellipsoids. **b,** Red and black lines indicate the east-west (EW) and north-south (NS) components, respectively, of the stacked GNSS time series (see Methods) at all stations except for those shown in Fig. 2. Light red and gray lines are the EW and NS components of the GNSS time series for each ETS.

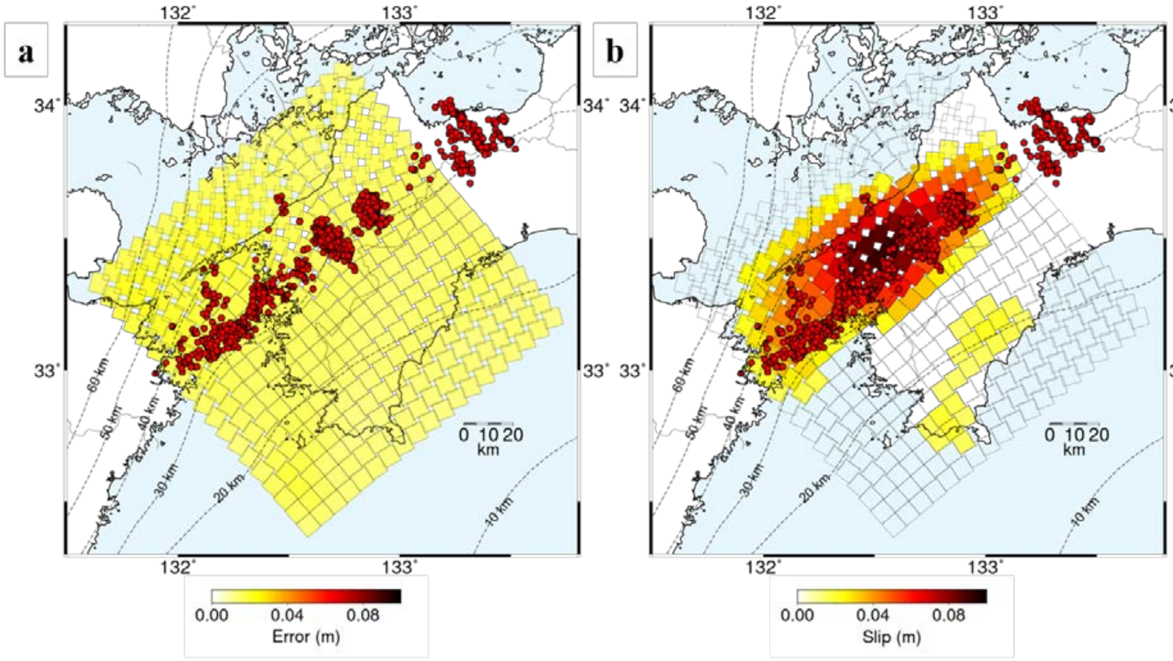

Fig. S2. Slip model and estimation errors. a, Distribution of the estimation error of fault slip shown in Fig. 3c. **b,** Slip distribution that exceeds the estimation error shown in **a**. Red dots indicate the LFE locations. The dashed lines are the depth contours of the upper surface of the subducting PH with an interval of 10 km[41-43]

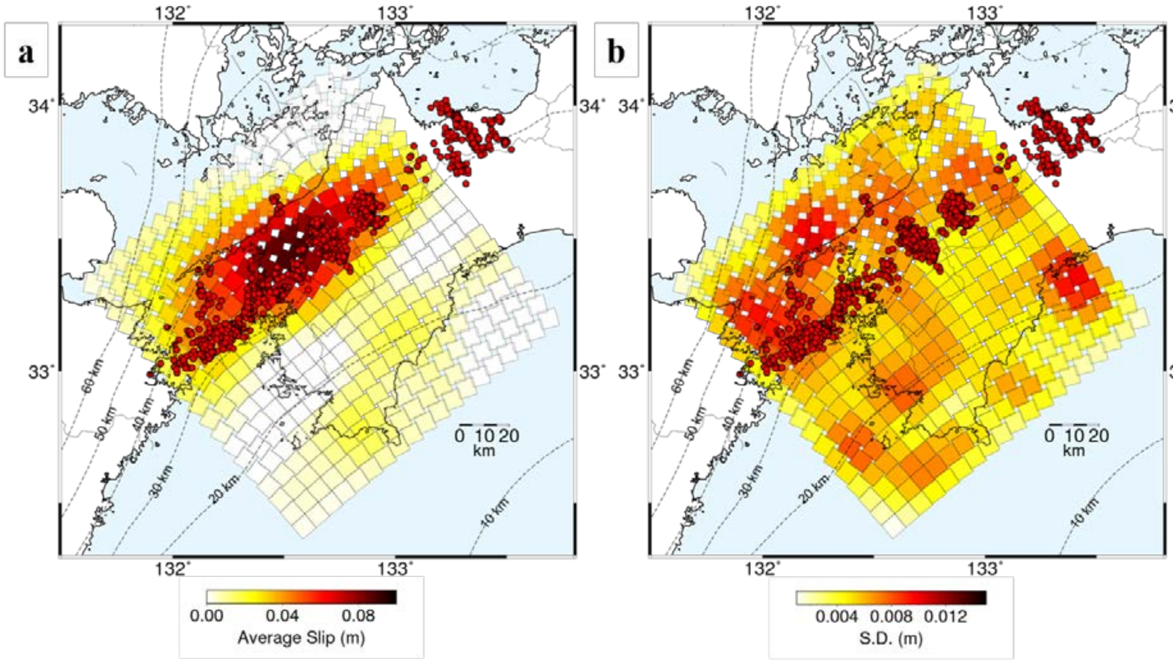

Fig. S3. Average slip model and its standard deviation using 31 stations. a, Slip distribution averaged for 100 cases obtained by using 31 randomly selected stations and **b,** its standard deviation. Red dots indicate the LFE locations. The dashed lines are the depth contours of the upper surface of the subducting PH with an interval of 10 km[41-43].

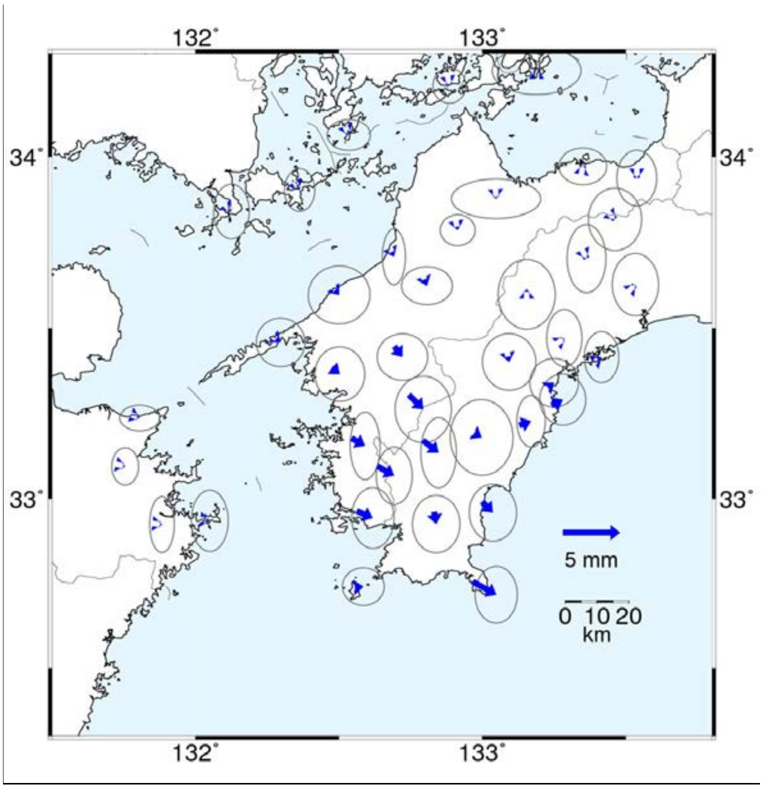

Fig. S4. Displacements due solely to slip in the shallower patch. Blue arrows indicate displacements calculated from slip only in the shallower patches with 2-σ observation errors.

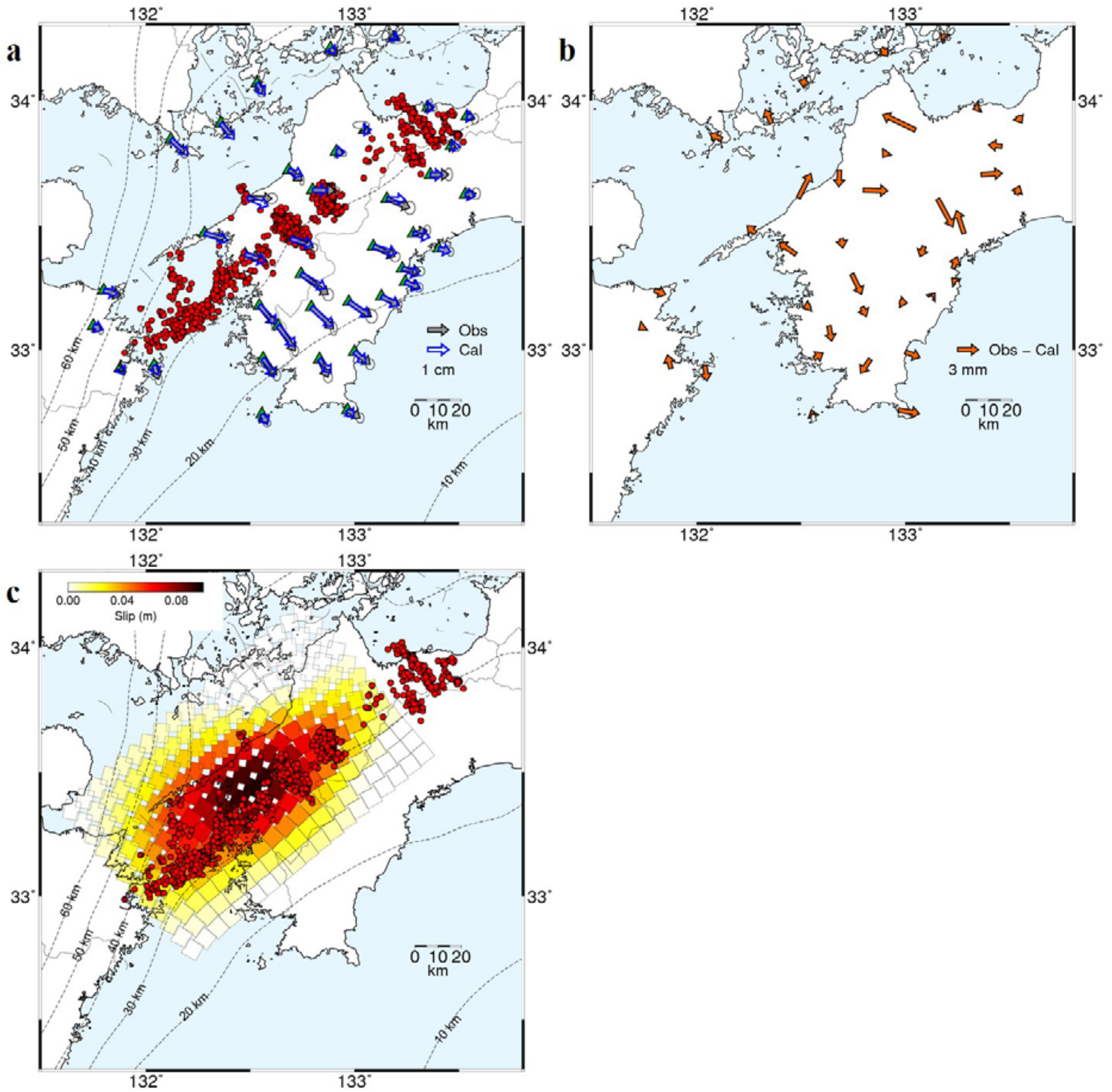

Fig. S5. Calculated and slip model without shallower subfaults. a, Comparisons between the observed (black) and the calculated (blue) displacement vectors. **b** the residuals between the two vectors. Ellipsoids indicate the 2-σ observation errors. **c,** Cumulative slip distribution. Red dots indicate the LFE locations. The dashed lines are the depth contours of the upper surface of the subducting PH at an interval of 10 km[41-43].

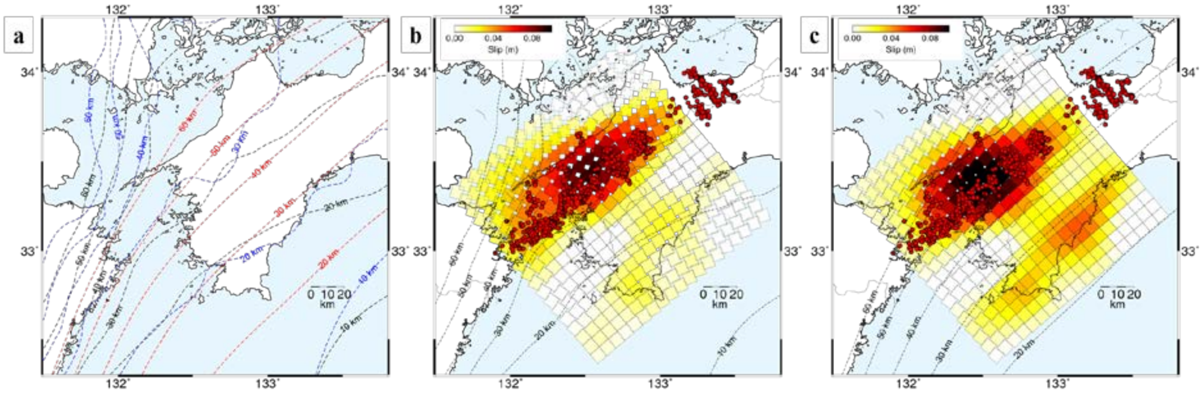

Fig. S6. Plate geometry and slip model. a, Comparison of the plate models. Black, red, and blue dashed lines are the depth contours of the upper surface of the subducting PH with an interval of 10 km obtained from refs. 41–43, Slab2[46], and ref. 47, respectively. We defined the upper surface of the PH as 10 km above the oceanic Moho geometry in the case of ref. 47. **b, c,** Slip distributions with smallest and largest slip amplitudes in the shallow slip patch among nine inversion cases (Table S2). Red dots indicate the LFE locations.

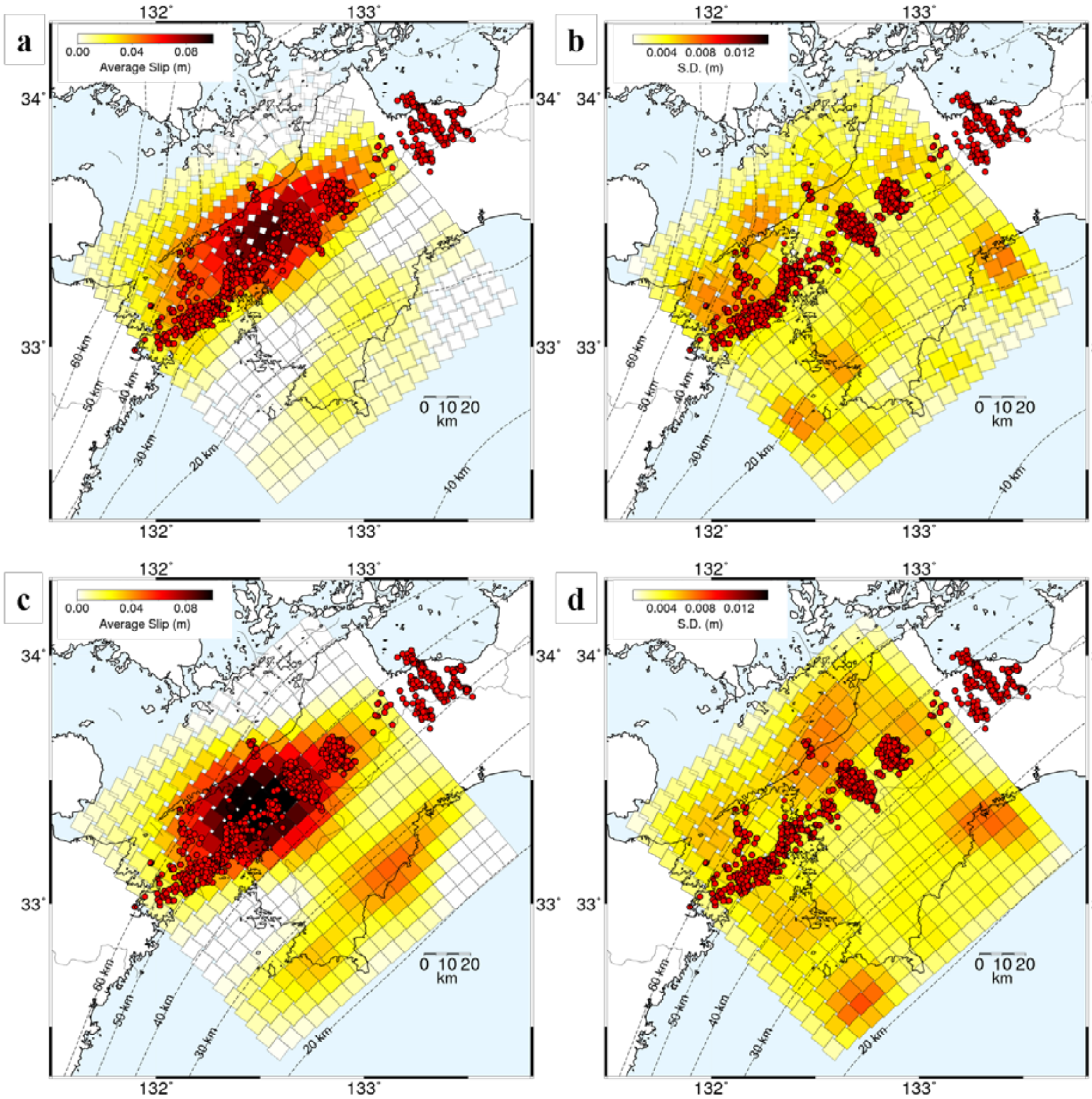

Fig. S7. Average slip models and their standard deviations in the cases of smallest and largest shallow slip using 35 stations. a, Slip distribution averaged for 100 cases obtained by using 35 randomly selected stations and **b,** its standard deviation in the case of smallest slip amplitude in the shallow slip patch shown in Fig. S6b. **c,** Slip distribution averaged for 100 cases obtained by using 35 randomly selected stations and **d,** its standard deviation in the case of largest slip amplitude in the shallow slip patch shown in Fig. S6c. Red dots indicate the LFE

locations. The dashed lines are the depth contours of the upper surface of the subducting PH (**a**,**b**[41-43]; **c**,**d**[46]) with an interval of 10 km.

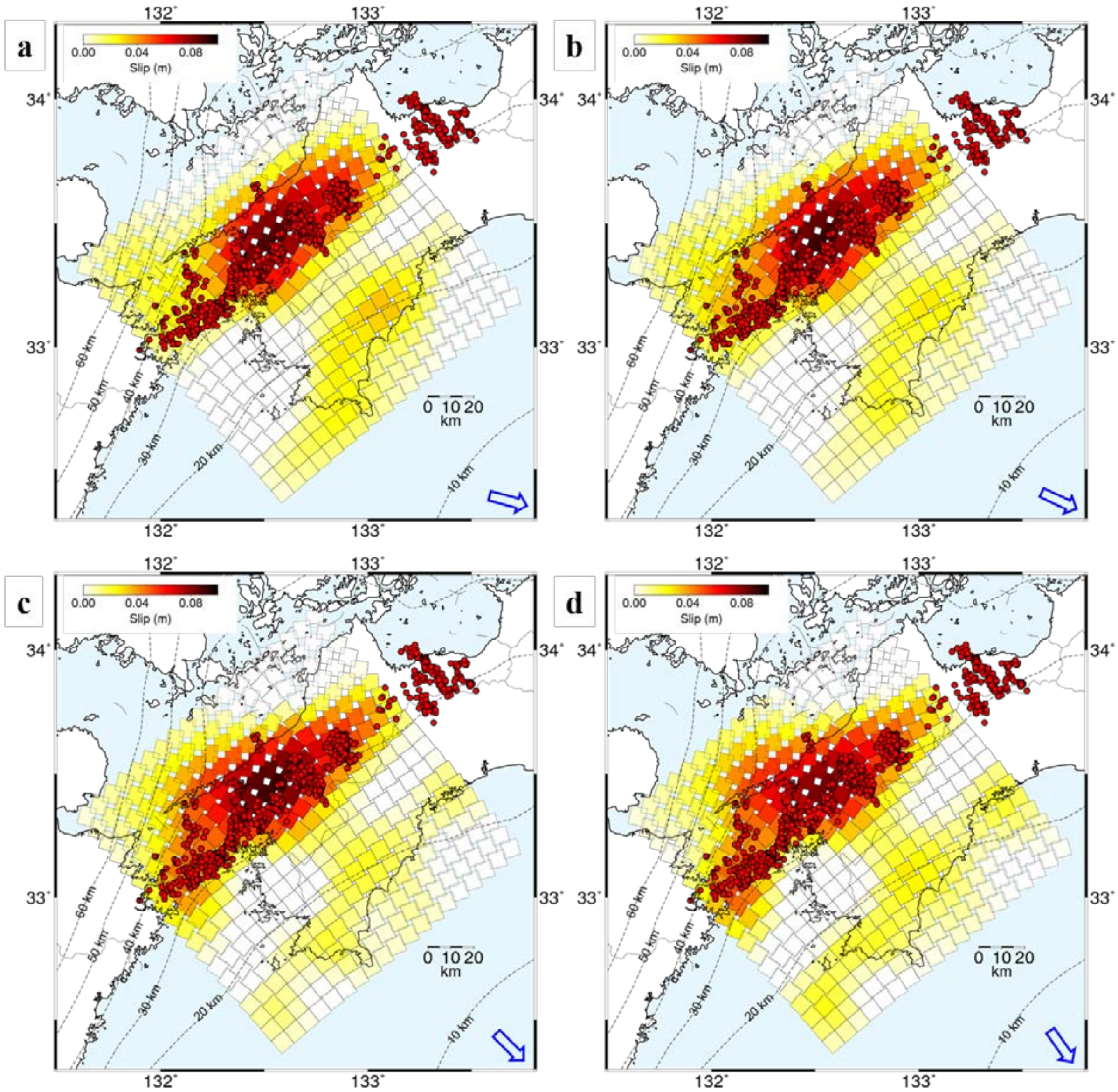

Fig. S8. Slip models with different slip directions. Slip distributions in cases of slip directions fixed to **a,** N105E, **b,** N115E, **c,** N135E and **d,** N145E, respectively. Note that the slip direction was assumed to be N125E[12] in the main result (Fig. 3) Red dots indicate the LFE locations.

The dashed lines are the depth contours of the upper surface of the subducting PH[41-43] with an interval of 10 km.

Table S1. List of S-SSEs in western Shikoku. Onset of S-SSEs detected by tiltmeters[16] and GNSS data[17] are summarized with their seismic moments, compared with the maximum day of LFE numbers.

Onset (tiltmeters[16])	Onset (GNSS[17])	LFE_max	M_o(x 10^{17} Nm)	
			tiltmeters[16]	*GNSS*[17]
2004/12/27	2004/12/29	2004/12/29	9.3	14.0
	2005/04/19	2005/04/17		17.0
2005/05/12		2005/05/20	17.7	
2005/10/23	2005/10/24	2005/10/26	5.1	11.0
	2006/01/21	2006/01/19		16.7
2006/04/15		2006/04/20	11.8	
2006/09/08	2006/09/17	2006/09/18	29.3	13.2
2007/03/13	2007/03/13	2007/03/14	4.7	14.1
2007/08/27	2007/09/09	2007/09/01	10.7	11.6
2007/12/19	2007/12/24	2007/12/20	19.9	18.0
2008/03/14	2008/03/25	2008/03/16	8.2	13.0
2008/09/27	2008/09/30	2008/09/29	9.2	14.4

Table S2. List of settings and results of inversion analysis. Seismic moments and their corresponding moment magnitudes summed up in the entire fault and the shallow fault exceeding the estimation error are summarized for each inversion case. *The inversion result shown in Figs. 3, S2 and S3 mainly discussed in the paper. **The inversion result shown in Figs. S6b, S7a and S7b exhibiting the smallest slip amplitude in the shallow slip patch. ***The inversion result shown in Figs. S6c, S7c, and S7d exhibiting the largest slip amplitude in the shallow slip patch.

Plate model	Poisson's ratio	M_o_all ($\times 10^{18}$ Nm)	M_w_all	M_o_shallow ($\times 10^{18}$ Nm)	M_w_shallow
Refs. *41-43*	0.20	13.0	6.68	0.91	5.90
	0.25*	13.1	6.68	0.86	5.89
	0.30**	13.5	6.69	0.85	5.89
Ref.*46*	0.20***	19.3	6.79	4.69	6.38
	0.25	19.8	6.80	4.52	6.37
	0.30	20.6	6.81	4.27	6.35
Ref.*47*	0.20	12.2	6.66	1.15	5.97
	0.25	12.3	6.66	1.19	5.98
	0.30	12.4	6.66	1.19	5.98
